# An improved method for studying mouse diaphragm function

**DOI:** 10.1038/s41598-019-55704-8

**Published:** 2019-12-19

**Authors:** Chady H. Hakim, Thais B. Lessa, Gregory J. Jenkins, Nora N. Yang, Carlos E. Ambrosio, Dongsheng Duan

**Affiliations:** 1 Department of Molecular Microbiology and Immunology, School of Medicine, The University of Missouri, Columbia, MO 65212 USA; 2 National Center for Advancing Translational Sciences (NCATS), Bethesda, MD 20892 USA; 3 Department of Veterinary Medicine, Faculty of Animal Science and Food Engineering, University of São Paulo, Pirassununga, SP 13635-900 Brazil; 4 Department of Biomedical, Biological & Chemical Engineering, College of Engineering, The University of Missouri, Columbia, MO 65212 USA; 5 Department of Neurology, School of Medicine, The University of Missouri, Columbia, MO 65212 USA; 6 Department of Biomedical Sciences, College of Veterinary Medicine, The University of Missouri, Columbia, MO 65212 USA

**Keywords:** Neurophysiology, Respiration

## Abstract

Dysfunction in the contractile properties of the diaphragm muscle contributes to the morbidity and mortality in many neuromuscular and respiratory diseases. Methods that can accurately quantify diaphragm function in mouse models are essential for preclinical studies. Diaphragm function is usually measured using the diaphragm strip. Two methods have been used to attach the diaphragm strip to the force transducer. The suture method is easy to adopt but it cannot maintain the physiological orientation of the muscle fibers. Hence, results may not accurately reflect diaphragm contractility. The clamp method can better maintain diaphragm muscle fiber orientation but is used less often because detailed information on clamp fabrication and application has never been published. Importantly, a side-by-side comparison of the two methods is lacking. To address these questions, we engineered diaphragm clamps using mechanically highly durable material. Here, we present a detailed and ready-to-use protocol on the design and manufacture of diaphragm clamps. Also, we present a step by step protocol on how to mount the diaphragm strip to the clamp and then to the muscle force measurement system. We compared the diaphragm force from the same mouse with both suture and clamp methods. We found the clamp method yielded a significantly higher muscle force. Finally, we validated the utility of the clamp method in the mdx model of Duchenne muscular dystrophy. In summary, the clamp method described in this paper yields reliable and consistent diaphragm force data. This method will be useful to any laboratory interested in performing mouse diaphragm function assay.

## Introduction

The diaphragm is a sheet-like muscle separating the thoracic cavity and the abdominal cavity. It is the primary muscle of inspiration. The contraction of the diaphragm expands the chest cavity, reduces lung pressure and leads to the inhalation of the air into the lung. The relaxation of the diaphragm allows the elasticity of the lungs and chest wall to push the air out of the lung. Diaphragm dysfunction-associated respiratory failure is a primary cause of morbidity and mortality in many neuromuscular and respiratory diseases. Numerous mouse models have been developed for these diseases. Accurate evaluation of the diaphragm force is essential in order to best utilize these models in preclinical studies.

**Figure 1:**
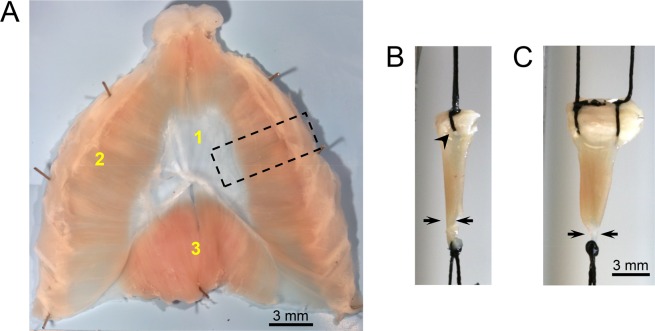
Mouse diaphragm and suture-based diaphragm strip mounting method. (**A**) A representative photomicrograph of a normal (BL10) mouse diaphragm viewed from the abdominal cavity. The mammalian diaphragm is composed of three components: 1. Central tendon; 2. Costal diaphragm; and 3. Crural diaphragm. The whole diaphragm muscle was dissected from a mouse and pinned in a Sylgard plate. Black dotted rectangle showing the region where the strip is cut out for force measurement. (**B**,**C**) Representative photomicrographs of two suture-based methods. (**B**) A single suture is used to hold the rib. (**C**) Two sutures are used to hold the rib. Black arrowhead showing a hole created by the suture in the diaphragm muscle in the one suture method. Black arrows showing how the diaphragm strip is pinched after tying a suture around the central tendon.

The mammalian diaphragm is composed of three components, the central tendon, costal diaphragm and crural diaphragm (Fig. 1A**)**. In mice, the central tendon is a semi-transparent membranous tissue. The costal diaphragm constitutes myofibers that are arranged radially from the central tendon to the lower rib cage. It is the primary inspiratory muscle. The dorsally located crural diaphragm inserts on the vertebrae instead of the rib cage. It provides a passage for the esophagus and aorta to the abdominal cavity.

Rodent diaphragm force measurement is usually performed using a muscle strip isolated from the costal hemidiaphragm **(**Fig. 1A**)**. This method was originally developed by Ritchie for studying rat diaphragm function^1^. The author isolated a 4-mm-wide costal diaphragm strip. The peripheral end of the strip had the rib and the other end had the central tendon. To attach the diaphragm strip to the force transducer, the author used a clamp to secure the rib and a suture to tie the central tendon. This method was subsequently adopted for studying mouse diaphragm function using muscle strips of ~1 to 5-mm-wide^2–4^.

Different methods have been used to secure the mouse diaphragm strip to the force transducer. These include stainless steel hooks^3^, sutures^2,5–7^, and clamps^8–10^. Since hooks may damage the diaphragm strip, this method is no longer used. Currently, the diaphragm strip force is measured using either the suture method or the clamp method. The suture method has been described in detail in the literature (www.treat-nmd.eu/downloads/file/sops/dmd/MDX/DMD_M.1.2.002.pdf)^5^. This method does not need special materials. However, the physiological orientation of the diaphragm muscle fiber is distorted **(**Figs. 1B and C**)**.

In order to maintain the diaphragm strip in its physiological shape, the clamp method was developed^8–10^. This method requires specially designed clamps to hold the rib and central tendon. Unfortunately, none of the published papers provided detailed information on the supplier, catalog number, the type and the size of the clamps^8–10^. No information is available on how the clamps are designed, manufactured, and used^8–10^.

**Figure 2:**
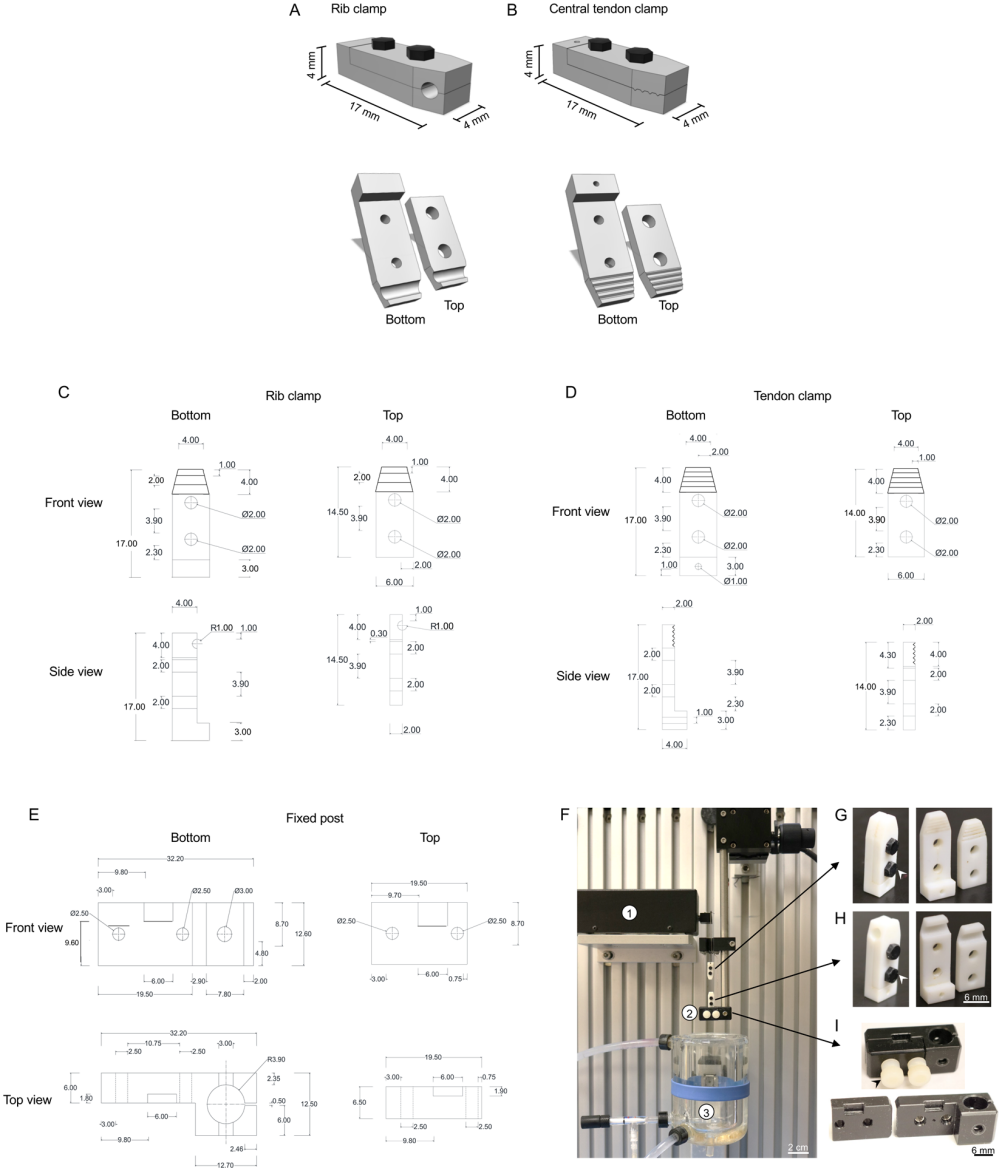
Diagrammatic illustration of the diaphragm strip clamps. (**A**) Top panel, the assembled rib clamp; Bottom panels, the top and bottom plates of the rib clamp. (**B)** Top panel, ﻿the assembled central tendon clamp;  Bottom panels, the top and bottom plates of the central tendon clamp. (**C**) A diagrammatic drawing of the rib clamp. (**D**) A diagrammatic drawing of the central tendon clamp. (**E**) A diagrammatic drawing of the fixed post. Measurements in panels C, D and E are in mm. (**F**) The fully assembled clamp assay system with clamps. (1) The servomotor transducer, (2) The fixed post for holding the body of the rib clamp, (3) The double jacket organ bath. (**G**) Left panel, the assembled central tendon clamp; Right panel, the individual clamp plates. (**H**) Left panel, the assembled rib cage clamp; Right panel, the individual clamp plates. White arrowhead, nylon hex screw used to assemble the two plates for  each clamp. (**I**) Upper panel, the assembled custom-designed fixed post to hold the rib cage clamp Bottom panel, the individual plate of the fixed post. Black arrowhead, nylon screw used to assemble the two plates of the fixed post.

To address these issues, we custom designed two sets of diaphragm clamps, one set for the rib cage and the other set for the central tendon **(**Fig. 2**)**. These clamps are not slippery and they can firmly hold the rib and central tendon during twitch, tetanic and eccentric contraction. Here, we provide detailed information on the design, manufacture, and use of these clamps. To determine whether the distortion of the diaphragm strip shape influences the outcome, we measured diaphragm force from the same mouse using the suture method and our newly designed clamp method. We found the clamp method yielded significantly higher muscle force when compared with the suture method. To establish the utility of the new clamp method for preclinical study, we compared diaphragm force between normal C57Bl/10 (abbreviated as BL10 in this paper) mice and dystrophic mdx mice. We demonstrated that the clamp method can reliably measure force deficits in the mdx diaphragm.

## Materials and Methods

### Experimental mice

All animal experiments were approved by the Animal Care and Use Committee of the University of Missouri and were in accordance with NIH guidelines. Dystrophin-deficient mdx mice and normal control BL10 mice were originally purchased from The Jackson Laboratory (Bar Harbor, ME). Experimental mice were generated by in-house breeding in a barrier facility using the breeders purchased from The Jackson Laboratory. The genotype of the mice was confirmed by PCR according to our previously published protocol^11^. Animal information is provided in the results section.

### Design of the diaphragm strip clamps

Two sets of clamps were designed by the author CHH **(**Fig. 2**)** to securely hold the diaphragm strip to the force transducer without imposing stress onto the muscle. The first set of clamps was designed to hold the rib and is named the “rib clamp” in this paper **(**Fig. 2A,C,H**)**. The second set of clamps was designed to hold the central tendon and is named the “tendon clamp” in this paper **(**Fig. 2B,D,G**)**. Each set of clamps is composed of a bottom plate and a top plate. The two plates are assembled together using two screws (Nylon hex head screw, 1/8′ width, 3/16′ long, 2–56 thread diameter size, McMaster-Carr, catalog number 99091A076, https://www.mcmaster.com/99091a076, Chicago, IL). At the tip of the rib clamp, a groove was designed to securely hold the entire length of the rib in the diaphragm strip without imposing stress on the muscle **(**Fig. 2A,G**)**. The size of the groove in the rib clamp was determined according to the anatomic circumference of the mouse rib to allow for easy fit. In the tendon clamp, the tip of the top and bottom plate remained flat but was v-grooved to allow a firm holding of the central tendon, without changing the physiological shape of the diaphragm strip. A hole was made at the distal end of bottom plate of the tendon clamp to tie a 4-0 suture loop. The loop connected to the force transducer lever arm via a stainless steel hook **(**Fig. 2F**)**. The weight of the stainless steel hook and the assembled tendon clamp are 0.03 g and 0.53 g, respectively. In our laboratory, we used the *in vitro* muscle apparatus from Aurora Scientific (Aurora Scientific, Inc., Aurora, ON, Canada) for force measurement. The fixed post of the muscle apparatus system is not compatible with our custom-designed diaphragm clamps. Hence, CHH made a custom-designed fixed post to hold the rib clamp **(**Fig. 2E,F,I**)**. The fixed post is made of two plates that are assembled together using a 10–32 Nylon screw (McMaster, Chicago, IL) **(**Fig. 2I**)**.

The prototypes of these designs were first produced using the 3D printing technology at the University of Missouri 3D Printing Facility (http://library.missouri.edu/printanything3d/). The final products were manufactured using polyethylene terephthalate, a material with excellent wear resistance and superior dimensional stability, at the University of Missouri Physics Machine Shop (University of Missouri, Columbia, MO).

### Diaphragm strip mounting plate

**Figure 3:**
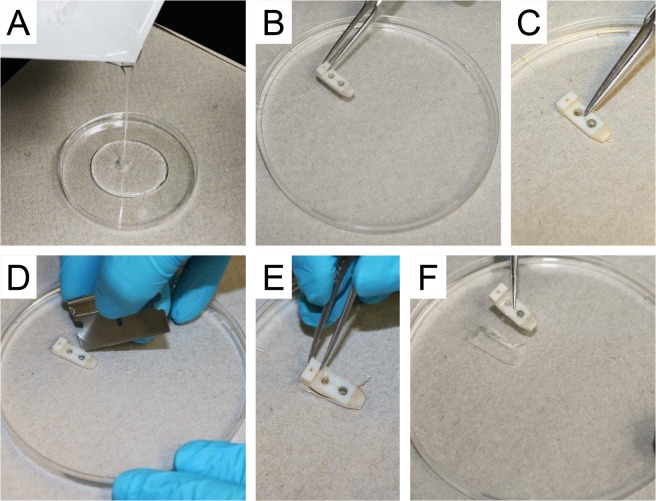
Diaphragm mounting plate. (**A**) Pouring the Sylagrd into a 120 mm petri dish. (**B**) Placing the bottom plate of the tendon clamp on the Sylagrd. (**C**) Submerging the bottom plate of the tendon clamp into the Sylgard. (**D**) Using a razor blade to dissect out the bottom plate of the tendon clamp. (**E**) Using a fine forceps to remove the bottom plate of the tendon clamp from the Sylgard. (**F**) The completed mounting plate with a template of the tendon clamp bottom plate engraved in the Sylgard.

Beside the clamps, we also made a diaphragm strip mounting plate with the Sylgard (Dow Corning, Midland, MI). Briefly, the Sylgard was prepared according to manufacture direction and poured into a 120 mm petri dish (USA Scientific, Ocala, FL) to a height of 2 mm (Fig. 3A**)**. Before the Sylgard was cured, the bottom plate of the tendon clamp was immersed in the Sylgard at the center of the plate (Fig. 3B,C**)**. Once the Sylgard was completely cured, the clamp plate was carefully removed leaving a permanent template-like shape of the bottom plate of the tendon clamp engraved in the Sylgard (Fig. 3D–F**)**.

### Diaphragm muscle dissection

Mice were anesthetized via intra-peritoneal injection of a cocktail containing 25 mg/ml ketamine, 2.5 mg/ml xylazine, and 0.5 mg/ml acepromazine at 2.5 µl/g body weight, then euthanized by cervical dislocation. Immediately after euthanization, the diaphragm was removed *en bloc* with the intact lower rib cage and rinsed with the 30 °C oxygenated (95% O_2_ - 5% CO_2_) Ringer buffer (pH 7.4; composition in mM: 1.2 NaH_2_PO_4_, 1 MgSO_4_, 4.83 KCl, 137 NaCl, 24 NaHCO_3_, 2 CaCl_2_, and 10 glucose) to wash off blood.

### Diaphragm strip preparation

**Figure 4:**
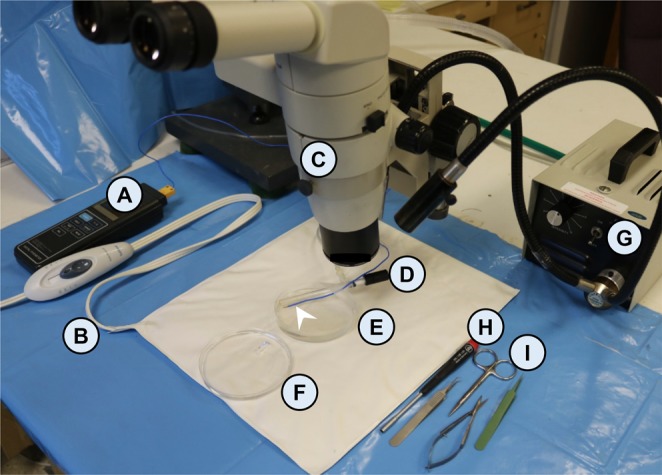
The diaphragm dissection tools. (**A**) Digital thermometer (B&K Precision Corporation, Yorba Linda, CA). (**B**) Heat pad (Sunbeam products INC., Boca Raton, FL). (**C**) Stereomicroscope (Nikon Instruments Inc., Melville, NY). (**D**) Oxygen bubbler (TNV Valves and Oxygen Disperser Tube, Radnoti, Covina, CA). White arrow head, thermocouple bead probe (B&K Precision Corporation, Yorba Linda, CA). The oxygen bubbler and the temperature probe are attached together to the dissection plate with a surgical tape (3M, St. Paul, MN). (**E**) Diaphragm dissection plate. (**F**) Clamp plate. (**G**) Fiber optic illuminator (Fiber-lite, Dolan-Jenner Industries, Boxborough, MA). (**H**) 1/8′ Hex screwdriver (McMaster, Chicago, IL). (**I**) Dissection tools: a pair of standard scissors, a pair of spring scissors, a pair of straight micro-forceps, a pair of 45° micro-forceps (Fine science tools, Foster City, CA).

**Figure 5:**
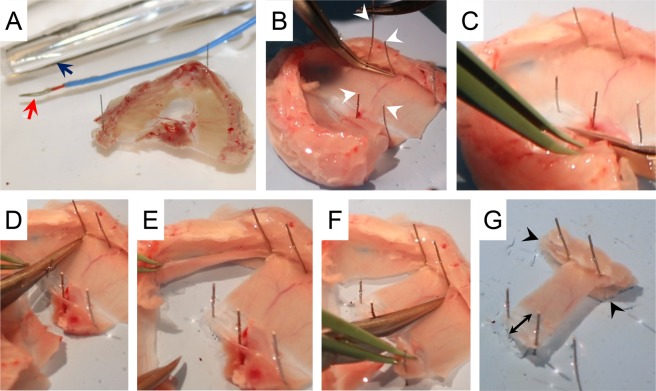
Preparation of the diaphragm strip for force measurement. **(A**) The diaphragm was dissected from the mouse and pinned in the dissection plate. Red arrow, temperature probe. Dark blue arrow, oxygenated bubbler (Radnoti LLC, Covina, CA). (**B**) Identification of the 4 mm diaphragm strip to be dissected using 4 pins. White arrowheads, pins used for marking the 4 mm diaphragm strip. (**C**–**G**) Dissection of the diaphragm strip. Black arrowheads, two lateral protruding ends of the rib. Double black arrow, the central tendon.

All tools needed to perform the diaphragm strip preparation are  presented in Fig. 4. After  removing the diaphragm muscle from the mouse, the diaphragm was transferred to the dissection plate **(**Fig. 4E**)**. The dissection plate is a 120 mm petri dish (USA Scientific, Ocala, FL) filled with the Sylgard (Dow Corning, Midland, MI) to a height of 5 mm. An oxygen bubbler (Radnoti LLC, Covina, CA) was placed on top of the Sylgard to oxygenate the Ringer buffer during the diaphragm strip dissection. Also, a thermocouple bead probe (B&K Precision Corporation, Yorba Linda, CA) was placed on the Sylgard to assure that the oxygenated Ringer buffer temperature was maintained at 30 °C throughout the dissection procedure. A heat pad (Sunbeam products Inc., Boca Raton, FL) was placed under the dissection plate to keep the buffer temperature at 30 °C during dissection.  The oxygen bubbler and  the temperature probe were  secured to the dissection plate with a surgical tape (3 M, St. Paul, MN) **(**Fig. 5A**)**.

The diaphragm muscle was pinned using 0.2 mm diameter stainless steel pins (Carolina Biological Supply, Burlington, NC) onto a transparent Sylgard block. The diaphragm muscle was covered with the oxygenated Ringer buffer throughout the dissection procedure. While viewing through a stereomicroscope (Nikon, Melville, NY), fat and connective tissues were carefully removed. Four pins were placed to mark a 4 mm wide diaphragm strip flanking the insertion of the phrenic nerve (~2 mm on each side) **(**Fig. 1A**)**. Two pins were placed at the rib cage site while the other two pins were inserted in the central tendon **(**Fig. 5B**)**. The 4 mm diaphragm strip was dissected just outside the 4 pins and along the long axis of the muscle fiber using a pair of fine forceps and a pair of sharp fine scissors (Fine Science Tools, Foster City, CA) **(**Fig. 5C–F**)**. The 4 mm strip included the two lateral protruding end of the rib and the central tendon **(**Fig. 5G**)**.

### Diaphragm strip mounting with the clamp method

**Figure 6:**
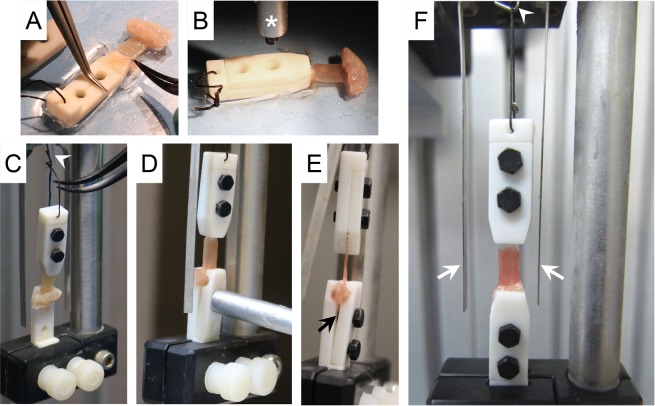
Step-by-step demonstration of the mounting of the diaphragm strip to the clamps and then to the muscle force system. (**A**,**B**) Mounting the central tendon to the tendon clamp. Asterisk, the hex wrench used to secure the clamp plates with the nylon hex screws. (**C**–**E**) Mounting the rib to the rib clamp and assembling the rib clamp. White arrowhead, stainless steel hook. Black arrow, a gap between the top plate and the bottom plate of the rib clamp. This is intentionally designed so that the rib clamp will not clamp on the diaphragm muscle after the clamp is assembled. (**F**) The final assembly of the diaphragm strip to the muscle force assay system using the clamp approach. White arrows, platinum electrodes.  White arrowhead, stainless steel hook.

To mount the central tendon, the bottom plate of the tendon clamp was placed in the custom-made Sylgard plate **(**Fig. 3F**)**. The whole central tendon area of the diaphragm strip was laid on top of the filleted surface at the tip of the clamp **(**Fig. 6A**)**. The top plate was secured to the bottom plate using two nylon screws **(**Fig. 6B**)**. Next, the assembled tendon clamp was attached to the stainless steel hook via a 4-0 suture **(**Fig. 6C**)**. The diaphragm strip was vertically positioned between two platinum electrodes **(**Fig. 6D–F**)**. The rib of the diaphragm strip was secured inside the rib clamp **(**Fig. 6D–E). The whole assembly with the diaphragm muscle strip **(**Fig. 6F**)** was submerged in a 30 °C jacketed organ bath (Radnoti LLC, Covina, CA) containing the oxygenated Ringer buffer. The muscle length was adjusted to generate a 1 g resting tension.

### Diaphragm strip mounting with the suture method

**Figure 7:**
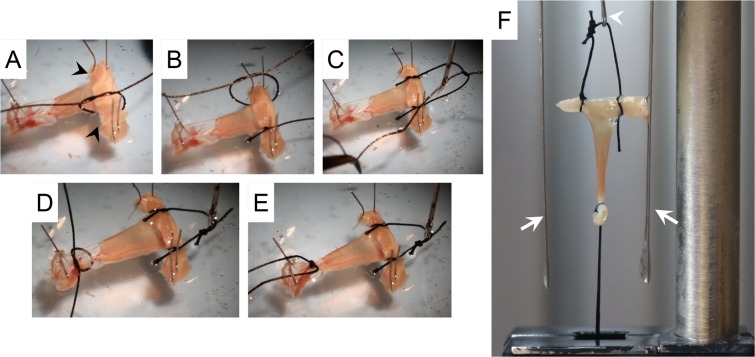
Step-by-step demonstration of the mounting of the diaphragm strip to the muscle force system using the suture approach. (**A**–**E**) Tying sutures to the protruding ends of the rib and the central tendon. Black arrowheads, the protruding ends of the rib. (**F**) Attachment of the diaphragm strip to the muscle force assay system using the suture approach. White arrows, platinum electrodes.  White arrowhead, stainless steel hook.

Diaphragm strip mounting was performed according to the published protocol as described in the TREAT-NMD web site (http://treatnmd.ncl.ac.uk/downloads/file/sops/dmd/MDX/DMD_M.1.2.002.pdf). Specifically, two 4-0 sutures were tied at the protruding ends of the rib and then the two sutures were tied together to form a loop **(**Fig. 7A–C**)**. Another 4-0 suture was tied around the central tendon near the muscle tendon junction **(**Fig. 7D,E**)**. The suture loop was attached to a stainless hook to connect with the force transducer. The central tendon suture was fixed in the fixed post **(**Fig. 7F**)**. The diaphragm strip was vertically positioned between two platinum electrodes **(**Fig. 7F**)**, and was submerged in a 30 °C jacketed organ bath containing the oxygenated Ringer buffer. The muscle length was adjusted to generate a 1 g resting tension.

### Diaphragm muscle force assay

Muscle force was evaluated at 30 °C in the Ringer buffer with a 305B dual-mode servomotor transducer (Aurora Scientific, Inc., Aurora, ON, Canada). The transducer was operated using the Dynamic Muscle Control software (DMC; version 3.12; Aurora Scientific). Data were analyzed using the Dynamic Muscle Analysis software (DMA; version 3.12; Aurora Scientific) and the DMA high throughput software (Version 2.0; Aurora Scientific). Exactly identical protocol was used to measure diaphragm contractility irrespective of the diaphragm mounting method (suture or clamp). However, due to the attachment of the tendon clamp to the transducer, we first balanced the transducer with the tendon clamp. Specifically, the assembled tendon clamp (without the diaphragm strip) was attached to the stainless-steel hook connected to the leveler arm and the weight of the clamp was tare (zero) using the DMC software. The electrical current was delivered by a pair of platinum electrodes flanking the diaphragm muscle strip **(**Figs. 6F and 7F**)**. After the diaphragm strip was transferred from the dissection plate to the tissue organ bath, it was allowed to equilibrate for 5 minutes. The diaphragm strip was then stimulated three times at 150 Hz with 1 minute rest between each stimulus to warm-up the muscle^12,13^. The optimal muscle length (Lo) was determined based on the isometric twitch force^14^. Briefly, twitch stimulation was applied while the muscle was strained at different lengths. The length that yielded the highest force was defined as Lo, and the muscle length was measured with an electronic digital caliper (McMaster, Chicago, IL). After the identification of Lo, we determined the optimal current and optimal stimulation duration. To determine the optimal current, first the muscle was set to Lo and stimulated at 1 Hz using 200 mA. Muscle force was recorded and analyzed following stimulation. Then, the muscle was re-stimulated after 60 seconds rest at a higher current and force was analyzed. The current was increased by an increment of 100 mA between each stimulus until muscle force reached the highest value and additional increase in the current resulted in force reduction. The current which produced the highest muscle force without inducing force drop was determined as the optimal current and used in the subsequent experiment steps. In our experimental setup, 800 mA consistently produced the maximal force without inducing muscle fatigue and was determined as the optimal current. To determine the optimal stimulation duration, the muscle was first stimulated at optimal Lo and current for 100 msec. The muscle force was recorded and analyzed. Then, the muscle was re-stimulated after 60 seconds rest at a longer stimulation duration and the force was analyzed. The stimulation duration was increased by an increment of 50 msec between each stimulus until muscle force reached the maximal stable plateau. Additional increase in the stimulation duration resulted in force reduction near the end of the stimulation. Thus, the duration which produced the maximal stable plateau was determined as the optimal stimulation duration and used in the subsequent experiment steps. In our experimental setup, 300 msec was sufficient to produce a maximal stable force plateau. The absolute twitch force (Pt) was measured at 1 Hz. The optimal maximal isometric tetanic force was determined by stimulating the muscle at different frequencies (5, 20, 40, 60, 80, 100, 120, 150 and 180 Hz), with 1 min rest between each stimulus. The highest force produced from these stimuli was considered as the absolute tetanic force (Po). After a 3 min rest, we determined the percentage of the force drop through 10 repetitive cycles of eccentric contraction. In each cycle the muscle was stimulated using the frequency that produced Po for 500 msec. After 300 msec stimulation, the muscle was stretched by 10% Lo at 0.5Lo/sec for 200 msec. The muscle was rested for 1 min between each cycle. At the end of the experiment, the diaphragm strip was gently removed from the clamps. The rib as well as the central tendon were  carefully dissected and the weight of the diaphragm strip was measured. The specific force (N/cm^2^) was calculated after normalizing the Po to the muscle cross-sectional area (CSA). The CSA was calculated according to the following equation, CSA = (muscle mass)/[(1.0 × optimal muscle length) × (1.06 g/cm^3^)]. 1.0 represents the ratio of the fiber length to the optimal muscle length (Lf/Lo) for the diaphragm muscle^6^. 1.06 g/cm^3^ is the muscle density.

In a different study, the diaphragm muscle force was evaluated in the presence and absence of d-tubocurarine **(**Supplementary Fig. 1**)**. The diaphragm muscle strip from 3-month-old male BL10 mice (n = 4) was prepared and mounted as described above. The twitch and tetanic forces were first determined in the absence of d-tubocurarine as described above. After the force values were recorded and analyzed, d-tubocurarine was added to the Ringer buffer at a final concentration of 30 µM^6,15^. After 5 min of incubation, the twitch and tetanic muscle forces were re-measured. Addition of d-tubocurarine did not significantly change the twitch and tetanic muscle forces **(**Supplementary Fig. 1**)**.

### Statistical analysis

Data are presented as mean ± standard deviation (SD). Statistical difference was determined by the Student t test for parametric data. The Mann-Whitney test was used for non-parametric data. *P* < 0.05 was considered statistically significant.

## Results

### The clamp method outperformed the suture method in force measurement

**Figure 8:**
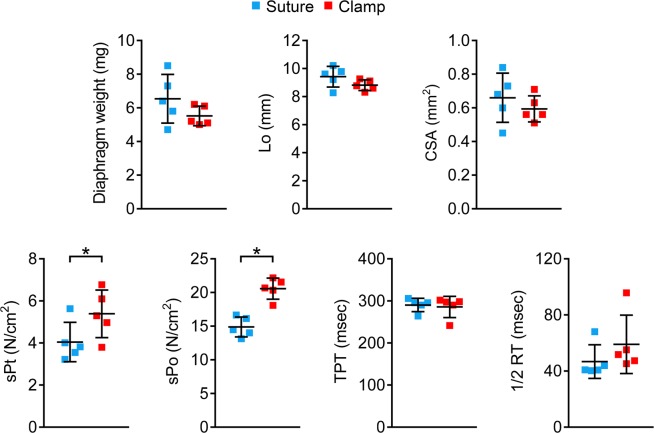
Comparison between the suture and clamp approaches. Quantitative comparison of diaphragm strip weight, optimal length (Lo), Cross sectional area (CSA), the specific twitch force (sPt), specific tetanic force (sPo), Time to peak tension (TPT), half relaxation time (1/2RT) between the suture and clamp approaches using diaphragm strips from 8-month-old male BL10 mice. Error bars are mean ± SD. Asterisk, clamp approach is significantly different from that of the suture approach.

To compare the clamp method and the suture method, we measured the twitch and tetanic force in 8-month-old normal male BL10 mice **(**Fig. 8**)**. To control for individual animal differences, we compared two methods using strips from the left and right hemidiaphragm of the same mouse. To further increase the stringency of the study, we alternated between the two methods and between the left-side and right-side strips. No significant difference was seen for the weight, optimal length and cross-sectional area between diaphragm strips used in two methods **(**Fig. 8**)**. However, the clamp method yielded a significantly higher specific twitch and tetanic force compared to that of the suture method **(**Fig. 8**)**. Nevertheless, the time to peak tension (TPT) and the half relaxation time (½ RT) were similar for the clamp method and the suture method.

### The clamp-based assay successfully detected muscle function deficiency in dystrophic mdx mice

To further validate our clamp method, we measured diaphragm force in 4-month-old male BL10 and mdx mice **(**Table 1**)**. Mdx mice are the most commonly used animal models for DMD, a lethal degenerative muscle disease^16^. It is well established that diaphragm muscle function is significantly compromised in mdx mice.

**Table 1:** Anatomic properties of the diaphragm strip in Fig. 9.

Strain	n	Weight (mg)	Lo (mm)	CSA (mm^2^)
BL10	8	4.69 ± 1.24	8.92 ± 0.58	0.50 ± 0.12
mdx	7	7.21 ± 0.83*	7.47 ± 0.35*	0.92 ± 0.11*

^*^Significantly different from that of the age-matched BL10 controls.

Values are means ± SD.

**Figure 9:**
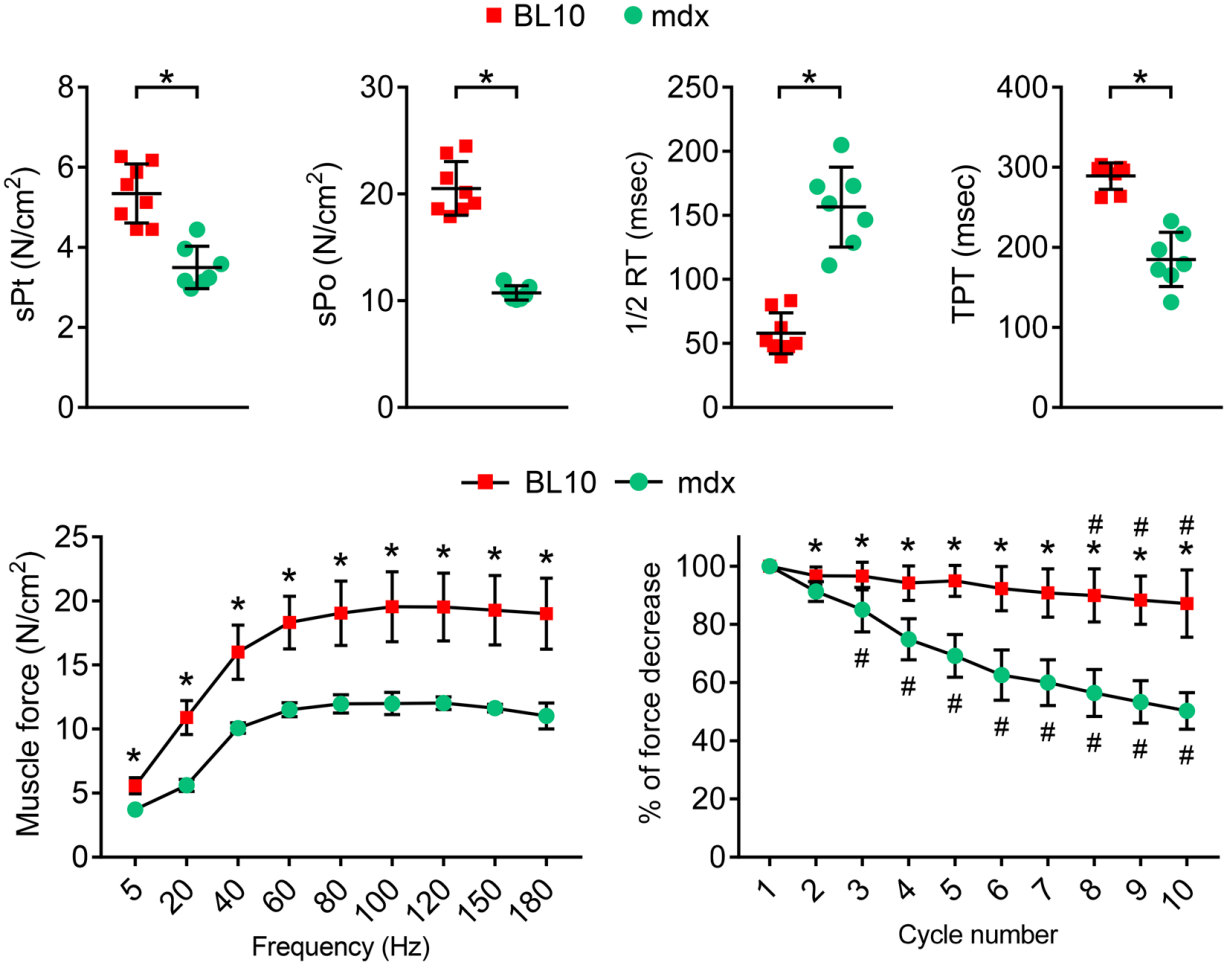
Evaluation of diaphragm function in 4-month-old male BL10 and mdx mice. Quantitative evaluation of muscle contractility and kinetics properties of the diaphragm muscle strip. Top panels: Specific twitch force (sPt), specific tetanic force (sPo), half relaxation time (1/2 RT) and time to peak tension (TPT). Bottom panels, muscle force at different stimulation frequency (5, 20, 40, 60, 80, 100, 120, 150 and 180 Hz) and time course of repeated cycles of eccentric contraction. Error bars are mean ± SD. Asterisk (*), mdx is significantly different from that of BL10. Pound sign (#), the % of force drop is significantly different from that of the first eccentric contraction cycle.

The diaphragm strip weight and cross-sectional area were significantly increased in mdx mice **(**Table 1**)**. Interestingly, the optimal muscle length was significantly decreased in mdx mice **(**Table 1**)**. The specific twitch and tetanic forces were significantly decreased in mdx mice **(**Fig. 9**)**. The force-frequency study further confirmed significant reduction of muscle force in the mdx diaphragm **(**Fig. 9, Supplementary Fig. 2**)**. Besides muscle force, we also examined the kinetics properties of the maximum tetanic force. The time to peak tension was significantly shorter in mdx mice, while the half relaxation time was significantly increased. Eccentric contraction is a sensitive assay often used to study muscle injury in DMD models. As expected, the mdx diaphragm was significantly more susceptible to eccentric contraction-induced muscle force drop **(**Fig. 9, Supplementary Fig. 3**)**.

## Discussion

In this study, we described and validated a novel clamp-based assay to study the mechanical function of the mouse diaphragm. The clamps were designed with the considerations of unique anatomic features of the mouse diaphragm **(**Figs. 1 and 2**)**. To allow easy adoption of our technique by other investigators, we provided detailed information on clamp manufacture, diaphragm dissection and diaphragm mounting **(**Figs. 2–6**)**. In a side-by-side comparison experiment, we found the clamp method yielded significantly higher specific twitch and tetanic forces than the suture method **(**Fig. 8**)**. We further showed that the clamp method reliably detected contractile deficiency in the dystrophic diaphragm **(**Fig. 9, Table 1, Supplementary Figs. 2 and 3**)**.

Diaphragm is a highly utilized muscle in the body. Diaphragm dysfunction leads to life threatening consequences. Ability to accurately quantify diaphragm force is a necessity in preclinical studies in a mouse model. The diaphragm strip can be mounted on the force transducer using either a suture or clamp approach. Two different suture methods have been described. In one method, the rib end of the diaphragm strip is tied with a single suture. Penetrating the suture through the rib is technically challenging and would likely damage the muscle fiber (www.treat-nmd.eu/downloads/file/sops/dmd/MDX/DMD_M.1.2.002.pdf) **(**Fig. 1B**)**. The problem encountered with the single suture method can be solved by using two sutures with each suture tied to one end of the rib **(**Fig. 1C**)**. Despite this improvement, the suture method still has a major drawback. Specifically, it is difficult to maintain a diaphragm strip in its physiological shape. This is largely because (a) the rib can be easily bent when sutures are pulled towards opposite directions during muscle contraction, and (b) the suture that ties the central tendon will inevitably squeezes nearby myofibers and alters their physiological organization. As a consequence, physiological orientation of the diaphragm muscle fiber is distorted. This deformation causes uneven force load on the diaphragm strip **(**Figs. 1B,C and 7**)**.

To determine whether the morphological change in the diaphragm strip can influence force measurement results, we compared muscle force between the suture and the clamp methods from the same mouse **(**Fig. 8**)**. To avoid bias, all the force assay was performed using the same instrument by the same investigator (TBL). We also rotated between the left and right side of the diaphragm strip and alternated the order of the diaphragm mounting method (suture or clamp). Although the suture method and clamp method yielded similar results for contraction kinetics (time-to-peak tension and half relaxation time), we found the specific twitch and tetanic forces were significantly reduced when diaphragm was mounted using the suture method. Our results suggest that distortion of diaphragm muscle fiber organization in the suture method indeed compromised force production.

It is worth pointing out that pathological changes (such as inflammation and fibrosis) are often not uniformly distributed in a diseased diaphragm. In these occasions, it is important to use a wide diaphragm strip for force measurement because data from a narrow muscle strip will less likely to reflect the disease in the whole diaphragm. However, for strips that were wider than 4 mm, it has been shown that the suture method resulted in artificial reduction of the specific muscle force because of the unnatural folding of the strip^5^. This will not be an issue with our novel clamp design, because the width of the clamps can be readily adjusted during manufacturing. In a preliminary study, we have successfully measured the force in diaphragm strips 6 to 8 mm wide.

The ultimate goal of our study is to apply the clamp method in preclinical studies. To this end, we compared the diaphragm strip force from age and sex-matched normal BL10 mice and dystrophin-null mdx mice **(**Fig. 9, Table 1, Supplementary Figs. 2 and 3**)**. The mdx mouse is a well-established animal model to study Duchenne muscular dystrophy. It has been shown by many groups that the force and kinetic properties of contraction are altered in the mdx diaphragm. Consistent with the literature, we found that the specific twitch force, specific tetanic force and time-to-peak tension were significantly reduced while the half-relaxation time was significantly increased in the mdx diaphragm. The force-frequency curve and eccentric contraction profile further demonstrated dysfunction of the mdx diaphragm **(**Fig. 9, Table 1, Supplementary Figs. 2 and 3**)**.

In summary, we reported the detailed design and manufacture information for making mouse diaphragm clamps. We also provided the step-by-step working protocol on using the clamp method to study mouse diaphragm function. Importantly, we showed superior performance of the clamp method compared to that of the suture method. We further showed that the clamp method can reliably detect diaphragm function deficits in a diseased diaphragm. Our method will enhance the reliability and reproducibility of the mouse diaphragm force assay.

## Supplementary information

Supporting Information

## References

[1] Ritchie JM (1954). The relation between force and velocity of shortening in rat muscle. J Physiol.

[2] Luff AR (1981). Dynamic properties of the inferior rectus, extensor digitorum longus, diaphragm and soleus muscles of the mouse. J Physiol.

[3] Supinski GS, Kelsen SG (1982). Effect of elastase-induced emphysema on the force-generating ability of the diaphragm. J Clin Invest.

[4] Stedman HH (1991). The mdx mouse diaphragm reproduces the degenerative changes of Duchenne muscular dystrophy. Nature.

[5] Moorwood, C., Liu, M., Tian, Z. & Barton, E. R. Isometric and eccentric force generation assessment of skeletal muscles isolated from murine models of muscular dystrophies. *J Vis Exp*, e50036, 10.3791/50036 (2013). 10.3791/50036 PMC3596910 23407283

[6] Lynch GS (1997). Contractile properties of diaphragm muscle segments from old mdx and old transgenic mdx mice. Am J Physiol.

[7] Petrof BJ (1995). Efficiency and functional consequences of adenovirus-mediated *in vivo* gene transfer to normal and dystrophic (mdx) mouse diaphragm. American journal of respiratory cell and molecular biology.

[8] Lopez MA, Pardo PS, Cox GA, Boriek AM (2008). Early mechanical dysfunction of the diaphragm in the muscular dystrophy with myositis (Ttnmdm) model. Am J Physiol Cell Physiol.

[9] Staib JL, Swoap SJ, Powers SK (2002). Diaphragm contractile dysfunction in MyoD gene-inactivated mice. American journal of physiology. Regulatory, integrative and comparative physiology.

[10] Mah C (2007). Physiological correction of Pompe disease by systemic delivery of adeno-associated virus serotype 1 vectors. Mol Ther.

[11] Shin J-H, Hakim C, Zhang K, Duan D (2011). Genotyping mdx, mdx3cv and mdx4cv mice by primer competition PCR. Muscle Nerve.

[12] Grange RW, Gainer TG, Marschner KM, Talmadge RJ, Stull JT (2002). Fast-twitch skeletal muscles of dystrophic mouse pups are resistant to injury from acute mechanical stress. Am J Physiol Cell Physiol.

[13] Hakim CH, Li D, Duan D (2011). Monitoring murine skeletal muscle function for muscle gene therapy. Methods Mol Biol.

[14] Hakim CH, Duan D (2013). Truncated dystrophins reduce muscle stiffness in the extensor digitorum longus muscle of mdx mice. J Appl Physiol.

[15] Cairns SP, Chin ER, Renaud JM (2007). Stimulation pulse characteristics and electrode configuration determine site of excitation in isolated mammalian skeletal muscle: implications for fatigue. Journal of applied physiology.

[16] McGreevy JW, Hakim CH, McIntosh MA, Duan D (2015). Animal models of Duchenne muscular dystrophy: from basic mechanisms to gene therapy. Dis Model Mech.

