# Supplementary material for: An improved method for studying mouse diaphragm function
Source: Sci Rep. 2019 Dec 19;9:19453. doi: 10.1038/s41598-019-55704-8 (PMC6923406; doi:10.1038/s41598-019-55704-8)
Supplement: Supplementary file 1 — Supporting Information [file 41598_2019_55704_MOESM1_ESM.docx]

**Supporting Information**

**An improved method for studying mouse diaphragm function**

Chady H. Hakim^1, 2, +^, Thais B. Lessa ^1, 3, +^, Gregory J. Jenkins^1,4^, Nora N. Yang ^2^, Carlos E. Ambrosio^3^, Dongsheng Duan^1, 4, 5, 6, *^

1, Department of Molecular Microbiology and Immunology, School of Medicine, The University of Missouri, Columbia, MO 65212, USA.

2, National Center for Advancing Translational Sciences (NCATS) Bethesda, MD 20892, USA.

3, Department of Veterinary Medicine, Faculty of Animal Science and Food Engineering, University of São Paulo, Pirassununga, SP, 13635-900, Brazil

4, Department of Biomedical, Biological & Chemical Engineering, College of Engineering, The University of Missouri, Columbia, MO 65212, USA.

5, Department of Neurology, School of Medicine, The University of Missouri, Columbia, MO 65212, USA.

6, Department of Biomedical Sciences, College of Veterinary Medicine, The University of Missouri, Columbia, MO 65212, USA.

^+^, both authors contributed equally

*Correspondence should be addressed to: Dongsheng Duan Ph.D.

Professor

Department of Molecular Microbiology and Immunology

One Hospital Dr.

Columbia, MO 65212, USA.

Phone: 573-884-9584

Fax: 573-882-4287

Email: [duand@missouri.edu](mailto:duand@missouri.edu)

Figure S1. Comparison of twitch and tetanic muscle force in the presence and absence of d-tubocurarine.

Figure S2. Comparison of diaphragm muscle force at different stimulation frequency in BL10 and mdx mice.

Figure S3. Percentage of force drop during eccentric contraction cycles in BL10 and mdx mice.

Figure S1. Comparison of twitch and tetanic muscle force in the presence and absence of d-tubocurarine.
